# Poxvirus Host Range Genes and Virus–Host Spectrum: A Critical Review

**DOI:** 10.3390/v9110331

**Published:** 2017-11-07

**Authors:** Graziele Pereira Oliveira, Rodrigo Araújo Lima Rodrigues, Maurício Teixeira Lima, Betânia Paiva Drumond, Jônatas Santos Abrahão

**Affiliations:** Laboratório de Vírus, Departamento de Microbiologia, Instituto de Ciências Biológicas, Universidade Federal de Minas Gerais, Belo Horizonte, Minas Gerais 31270-901, Brazil; graziufmg@yahoo.com.br (G.P.O.); rodriguesral07@gmail.com (R.A.L.R.); maurili15@hotmail.com (M.T.L.); betaniadrumond@gmail.com (B.P.D.)

**Keywords:** *Poxviridae*, network, host range genes, horizontal gene transfer, evolution

## Abstract

The *Poxviridae* family is comprised of double-stranded DNA viruses belonging to nucleocytoplasmic large DNA viruses (NCLDV). Among the NCLDV, poxviruses exhibit the widest known host range, which is likely observed because this viral family has been more heavily investigated. However, relative to each member of the *Poxviridae* family, the spectrum of the host is variable, where certain viruses can infect a large range of hosts, while others are restricted to only one host species. It has been suggested that the variability in host spectrum among poxviruses is linked with the presence or absence of some host range genes. Would it be possible to extrapolate the restriction of viral replication in a specific cell lineage to an animal, a far more complex organism? In this study, we compare and discuss the relationship between the host range of poxvirus species and the abundance/diversity of host range genes. We analyzed the sequences of 38 previously identified and putative homologs of poxvirus host range genes, and updated these data with deposited sequences of new poxvirus genomes. Overall, the term host range genes might not be the most appropriate for these genes, since no correlation between them and the viruses’ host spectrum was observed, and a change in nomenclature should be considered. Finally, we analyzed the evolutionary history of these genes, and reaffirmed the occurrence of horizontal gene transfer (HGT) for certain elements, as previously suggested. Considering the data presented in this study, it is not possible to associate the diversity of host range factors with the amount of hosts of known poxviruses, and this traditional nomenclature creates misunderstandings.

## 1. Introduction

Poxviruses are among the best known and most feared viruses. The *Poxviridae* family is currently divided in two subfamilies, named *Entomopoxvirinae* (insect-infecting viruses) and *Chordopoxvirinae* (vertebrate-infecting viruses), wherein the first is composed of three genera, and the latter contains 10 genera, in addition to two viral species that have yet to be classified into each subfamily [1]. While the entomopoxviruses have been poorly investigated over the years, the chordopoxviruses are among the most studied groups in virology, due to the medical and veterinary relevance of many of their members. Among the chordopoxviruses, the *Variola virus* (VARV_abbreviations are shown in Supplementary Table S1) is one of the most well-known species. VARV is the agent of smallpox, a disease that has plagued humanity for centuries, until it was considered eradicated by the World Health Organization in 1980 after a successful global vaccination and surveillance campaign [2,3,4]. Other chordopoxviruses, such as vaccinia virus (VACV), cowpox virus (CPXV) and monkeypox virus (MPXV), are responsible for several outbreaks of exantematic diseases around the world, both in humans and other animals (e.g., bovines and equids), and are considered emergent zoonotic viral diseases [5,6]. Furthermore, studies with poxviruses have been pivotal for the advancement of other areas of knowledge, especially in cell biology, vaccinology, and virotherapy, where it was possible to elucidate many important metabolic pathways for immune response and the development of different strategies of immunization against infectious diseases [7,8,9,10].

The *Poxviridae* family consists of large double-stranded DNA viruses, which replicate entirely in the cytoplasm of host cells [11]. The poxviruses have a complex structure and an extensive linear genome ranging from 128 to 365 kbp (Genera *Parapoxvirus* and *Avipoxvirus*, respectively), which code for over 200 genes [12,13,14]. *Chordopoxvirinae* is divided in two clusters that are well-resolved phylogenetically. The first of the two clusters corresponds to the *Orthopoxvirus* (OPV) genus. The second cluster formed by the genera *Yatapoxvirus*, *Leporipoxvirus*, *Capripoxvirus*, *Cervidpoxvirus,* and *Suipoxvirus* forms a sister clade to orthopoxviruses, and the former can be classified as “clade II” poxviruses [15,16]. The origin and evolution of poxviruses are still blurred. Although there is some strong evidence suggesting that these viruses emerged thousands of years ago, their genome has evolved through the gain and loss of genes, especially through gene duplication and horizontal gene transfer (HGT) [15,16,17]. Many of the genes present in the poxvirus genome are not essential to viral replication in cell culture, but are important to the modulation of the host antiviral response, and thus are considered virulence genes [18,19]. Some of these genes impact viral replication only in a set of cell lineages that originated on different tissues or host species. These genes act on poxvirus-specific differences in tropism and host range, and have been referred to as host range genes [18,19,20]. All poxviruses are predicted to encode a unique collection of host range genes; however, only the genera *Orthopoxvirus* and *Leporipoxvirus* have been observed in many of the biological studies so far [13]. Known poxvirus host range genes are currently grouped into 12 distinct classes, some of which have only one gene (e.g., K3L, E3L, K1L, others), and others exhibiting many members (e.g., serpins, C7L family, TNFRII family, others), which likely result from lineage duplication events [20]. Some of these factors were functionally characterized using in vitro models and gene knockout analysis, which is associated mostly with the manipulation of diverse cellular targets, including cellular kinases and phosphatases, apoptosis, and many antiviral pathways [19,21]. In the absence of these genes, viruses lose the ability to infect certain cell lineages, whereas infection is efficiently established in the presence of the genes. Several in vivo investigations showed that some factors impact viral pathogenicity, although the model animals were still infected [22,23,24].

Historically, these genes have been referred to as host range genes when considering only the cells as hosts, i.e., not considering the animals that are actually infected by the viruses [18,19,20]. Would it be possible to extrapolate the restriction of viral replication in a specific cell lineage in an animal, which is a far more complex organism? Some works have suggested a direct association between the diversity of host range factors and the amount of host species for different poxviruses, but this association is still under debate [18,19]. In view of these intriguing questions, we sought to establish the natural hosts for the poxviruses officially assigned to viral species and recognized by the International Committee on Taxonomy of Viruses (ICTV) [1]. Based on the available data so far, we performed an extensive search for different host range genes according to those that were described previously [20], reviewing the main features of each class of host range factors. In light of the data presented here, we could not associate a diversity of host range factors with the amount of hosts, which lead us to discuss the assertiveness of the term “host range genes”. Finally, we analyzed the evolutionary history of these genes, reaffirming the occurrence of HGT for some elements, as previously suggested [16,17,20].

Among the nucleocytoplasmic large DNA viruses (NCLDV), the poxviruses are those with the widest host range, which are able to infect different groups of insects and vertebrates [25]. However, when we look specifically at each member of the *Poxviridae* family, the host spectrum is variable, wherein some viruses can infect a large range of hosts [25,26,27], while others are restricted to only one host species [3,28,29]. To have a clear view of the host range of poxviruses, we performed an extensive search to define the natural hosts for each member of the *Poxviridae* family, and we presented this relationship in a network graph. In this analysis, we searched only the hosts for viruses that are currently classified into viral species by the ICTV, since it presents the most up-to-date dataset of known viral species, and gathers and reflects the diversity of the circulating viruses in nature. We defined hosts as those organisms in which consistent evidence was available related to viral detection in a given species by isolation, serology, and molecular detection. In this view, we seek to associate hosts at the lowest possible taxonomical level.

**Figure 1:**
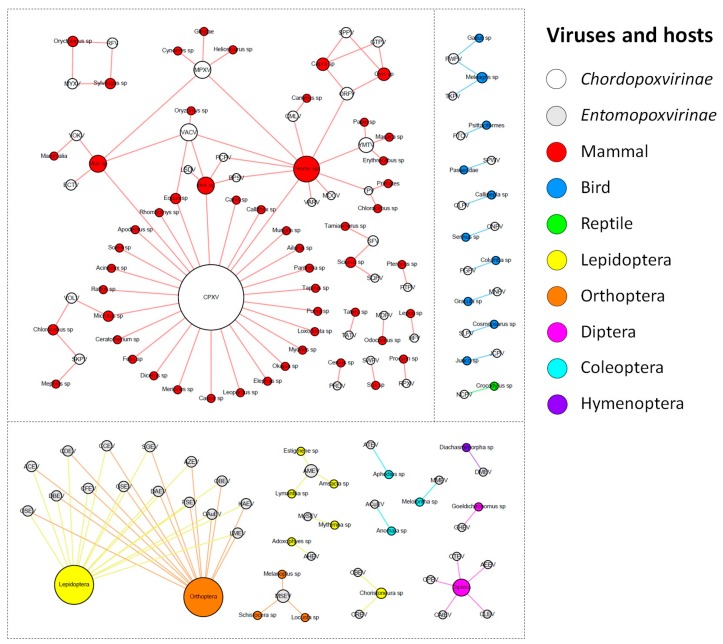
Poxvirus host network. The poxvirus species and their hosts (taxonomic level of genus or higher) are depicted in the graph. The network layout was generated by applying a force-based algorithm, followed by manual rearrangement of the nodes. The full names of the viral species are listed in Supplementary Table S1.

The ICTV currently recognizes a total of 71 species of poxvirus, with 30 belonging to the *Entomopoxvirinae* subfamily, and 41 to the *Chordopoxvirinae* subfamily (Supplementary Table S2). The known entomopoxviruses infect Pterygota subclass members (winged insects) from the orders Diptera, Coleoptera, and Hymenoptera, but mainly Lepidoptera and Orthoptera (Figure 1).

For most of the viral species of this group, it was not possible to determine the virus hosts beyond the order taxonomic level. For the remaining viral species, we determined the hosts at the genus or species level. Corroborating the previous descriptions in the literature, entomopoxviruses tend to exhibit a fairly narrow host range, but the species of *Betaentomopoxvirus* genus can infect distant hosts, which suggests that large host shifts can occur. This view of entomopoxvirus hosts is likely a consequence of the lack of host range studies that have been performed on these viruses [30,31]. Recently, a study showed that genes involved in lateral gene transfer (LGT) events among entomopoxviruses species conferred a possible adaptation to both specific and distantly related hosts [32]. It is possible that with the advancement of metaviromic approaches, we will be able to move forward in our comprehension of the diversity and host range of entomopoxviruses, uncover new viruses in different and unexplored hosts, and therefore improve the network presented here [33].

In contrast, the chordopoxviruses are the targets of intense investigation because of the clinical relevance of many of its members to humans and domesticated animals of economic importance [34,35,36,37,38]. The known chordopoxviruses infect mammals (29 viral species), birds (10 viral species), and reptiles (one viral species), and viruses do not cross this host barrier, i.e., viruses infecting mammals do not infect birds, and vice versa (Figure 1). Interestingly, an avipoxvirus was isolated once from a terminally ill rhinoceros in 1969 and characterized as an atypical fowlpox virus, thus raising questions about the host restriction of avipoxviruses [39]. However, since it was an isolated case and there are no other descriptions of an avipoxvirus infecting a non-avian host, it is still uncertain whether these viruses can efficiently cross the host barrier. Differently from entomopoxviruses, there were only four chordopoxviruses for which we could not define the hosts at the genus or species level (4/40 = 10%). One of these viruses is the yokapox virus (YOKV), a poxvirus isolated from a mosquito pool over 40 years ago whose natural host is probably a mammal. However, further investigation is required to better understand the biology of this virus [40]. Similar to YOKV, it is possible that other hosts are associated with known chordopoxviruses, but these relationships need to be further investigated. Most of the chordopoxviruses are associated with only one host genus (25/40 = 62.5%), which suggests a restricted host range for these viruses (Figure 1). Interestingly, there is a trend among large DNA viruses to exhibit a narrow host range, but some viruses can infect a broader range of hosts, such as VACV and MPXV, which have been associated with outbreaks involving humans and cattle. For these viruses, it is likely that rodents act as reservoir hosts of these viruses [41,42,43]. The CPXV is the most prominent member in the group, since it presents by far the widest host spectrum among the poxviruses, being able to infect at least 27 different groups of hosts, including humans, cattle, equids, and felines (Figure 1). Recent data suggests that *Cowpox virus* is comprised of at least five different viral species [44]. Assuming this, it is likely that the hosts defined here of what we now consider as *Cowpox virus* are actually a host compilation of hosts for at least five distinct viral species.

After the eradication of smallpox in 1980, several poxvirus outbreaks have been reported around the world that are related to different viruses, driving a huge effort to identify these viruses and their possible natural and reservoir hosts [45,46,47,48]. At least 11 poxvirus species have been known to cause human infections to date: CPXV, VACV, MPXV, VARV, *Molluscum contagiosum virus* (MOCV), *Orf virus* (ORFV), *Camelpox virus* (CMLV), *Yaba monkey tumor virus* (YMTV), *Tanapox virus* (TPV), *Bovine papular stomatitis virus* (BPSV), and *Pseudocowpox virus* (PCPV) [49,50] (Figure 1). Among them, only VARV and MOCV have humans as the sole host, while the other poxviruses are emerging zoonoses that affect different groups of animals, such as cattle, rodents, primates, and others (Figure 1) [3,28]. The main clinical feature of poxvirus infection is skin lesions, which can vary from small pearly papules in MOCV infection to large crusts and generalized pustules in VARV infection. However, other symptoms are common, including fever, headache, and rash. Similar clinical signals are verified in other animals infected by these viruses. Cases of human infections by some poxviruses, such as VACV, CPXV, and MPXV, have been constantly reported [3,26,28,35,51]. Differently, skin lesions related to CMLV have only been observed during the last few years, which opens important questions regarding the expanding host range of this virus [52]. The *Parapoxvirus* genus comprises four viral species that are distributed worldwide and mainly infect domestic ruminants and a broader host range, which includes camels, seals, deer species, and humans [53]. In this context, it is possible that other known poxviruses might affect humans. An example of this is the isolation of a strain of *Ectromelia virus* (ECTV) from the throat swabs of an affected man in China during an outbreak of erythromelalgia [54]. It is still uncertain whether this virus is a true human pathogen, since ECTV has only been found in *Mus* sp. (Figure 1), causing mousepox disease [55]. If this association is confirmed, it will be another example of host range expansion in poxviruses. Furthermore, one must have in mind that a virus can infect a host without causing any disease. Although the majority of poxviruses isolation reports are related to any clinical manifestation in a given host, some viruses have already been identified in known hosts without presenting any clinical manifestation, such as the group 1 of Brazilian VACV strains [41]. Moreover, there are descriptions of poxvirus detection in cattle, but the hosts had no clinical signals [56]. There is also a description of the isolation of a parapoxvirus from an apparently healthy red deer in the Bavarian Alps [57], which reinforces that poxviruses are related to hosts exhibiting no disease. In this context, we might expect that the network presented here is likely more interconnected, but more recurrent data regarding poxvirus’ detection in healthy organisms will be needed.

Based on currently available data, it appears that certain poxviruses have a broad host range, such as VACV, MPXV, and CPXV. However, these viruses are the most studied within the *Poxviridae* family, which is the reason why we recognize more host species for them compared with other viruses, and especially compared with entomopoxviruses. In contrast, other viruses that are also the targets of intense research exhibit a very restricted host range, as is the case with VARV and MOCV (both infect only humans). The reason for such a difference is still a mystery, but some studies have suggested that the answer might lie in the genetic diversity between those viruses, namely, the diversity and abundance of the referred host range genes [18,19].

## 2. Diversity and Abundance of Host Range Factors Are Not Proportional to the Diversity of Hosts in *Poxviridae*

Host range genes are virulence genes of poxviruses, which code for factors that influence the virus’ tropism for certain cell lineages [18]. Historically, the first of these genes to be described belonged to the serpin superfamily (serine protease inhibitors), which was initially found in the rabbitpox virus over 60 years ago, where it was verified in “white pock” mutants observed in the chorioallantoic membrane. It was associated with the viral loss of the ability to replicate in some cell lineages, and posteriorly mapped to the SPI-1 gene [58,59,60,61,62]. Thereafter, other genes that impacted the replication of poxviruses in some cell types were described, reiterating the definition of “host range genes” [21].

There are currently 12 groups of known host range factors. These factors are made up of a few dozen genes that are heterogeneously distributed within the *Poxviridae* family, and no single viral host range ortholog common to all poxvirus genomes has been identified [19,20,21]. Traditionally, the discovery of these genes has been the result of in vitro analysis, with targeted knockout genes in which mutant viruses exhibited deficiencies in replication in a subset of cells that are permissive to parental viruses [63,64]. In contrast, in vivo assays have shown that the absence of these genes does not impair viral infection in the animal model, but does affect the degree of pathogenicity of the virus, even though in vivo assays are lacking for the majority of the host range genes [65,66]. With the advancement of molecular techniques, some genes in different poxviruses have been suggested as host range genes due to their homology compared with other previously described genes, i.e., K3L, C7L and m63r, E3L, M11L/F1L, ANK-F-box CP77, and T5, but the function of the putative genes lacks experimental validation [20].

The function of some host range genes is already known, but some functions remain obscure. Generally speaking, the products of these genes interact with antiviral and/or anti-inflammatory host pathways, including the interferon (IFN), apoptosis, and inflammasome pathways [21]. Among the known and predicted host range genes found in poxviruses, the most studied are the E3L and K3L VACV genes. E3L encodes a protein that contains a C-terminal double-stranded RNA-binding domain, which acts as an inhibitor of protein kinase R (PKR) and 2′-5′ oligoadenylate synthase, likely by preventing activation by dsRNA and induction by IFN [67,68,69,70]. Similar to E3L, the K3L protein also acts by inhibiting the PKR activity, since it is homologous to the S1 domain of the α subunit of eukaryotic translation initiation factor 2 (eIF2), therefore acting as a pseudosubstrate and competitive inhibitor of the kinase protein [71,72,73,74,75]. Another well-known host range gene is SPI-1, but it is possible that other serpins also contribute to poxvirus host range genes, including *Orthopoxvirus* SPI-2/CrmA and SPI-3, three serpins in the *Leporipoxvirus* genus, and five serpins in the *Avipoxvirus* genus [76]. The serpins in the poxvirus counteract the host response to viral infection, acting on different targets of inflammation, coagulation, and complement activation pathways [76]. Some poxviruses also present genes coding for proteins containing short complement-like repeats (SCRs)—the B5R/VCP family—which interacts with a yet to be identified cellular surface molecule that leads to the activation of Src kinase, prompting actin polymerization and the enhancement of cell-to-cell virus spread [77,78].

Poxviruses have many proteins with ankyrin repeat domains (ANK), most of which contain an F-box domain at their carboxy-terminal region. A poxvirus ANK protein has been identified as a host range factor, named CP77 or CHOhr, since its absence in VACV led to a restriction of replication in a Chinese hamster ovary cell line [79]. At the molecular level, CP77 inhibits nuclear factor ΚB (NF-ΚB) activation, likely by the interaction with the ANK repeats in the p65 subunit of NF-ΚB and the F-box of the cellular SCF ligase complex [80]. Other ANK proteins might have similar functions, but further evidence is required. Similar to these, other genes/families of genes have already been identified as host range genes, but their molecular mechanism is not fully elucidated, such as p28/N1R, and the C7 family. The C7 family contains C7L and C4L, which were initially identified in the VACV genome, and other three C7L-related genes that were found in leporipoxviruses, M062R, M063R, and M064R [20,81].

Classes of host range genes were initially identified in leporipoxviruses, and their presence was further described in other poxviruses [21]. The T2 gene found in *Myxoma virus* (MYXV) is a homolog of tumor necrosis factor receptor II (TNFRII), in which the resulting protein acts by neutralizing the host TNF, thus impairing the inflammation response [82,83]. Homologs of T2 are found in some orthopoxviruses, in which they are called cytokine response modifiers (crm) [20,84], and comprise the TNFRII family of host range genes. The T4 and M11L/F1L are families of genes that inhibit apoptosis and were initially described in MYXV and VACV, but the precise molecular mechanism is not completely clear [20,85]. Finally, the M13L gene found in MYXV contains an N-terminal pyrin domain, while the M1 in MYXV was shown to inhibit caspase-1 activation and thus the processing of mature IL-1β and IL-18 in human THP-1 cells, impairing the inflammatory response [86].

**Figure 2:**
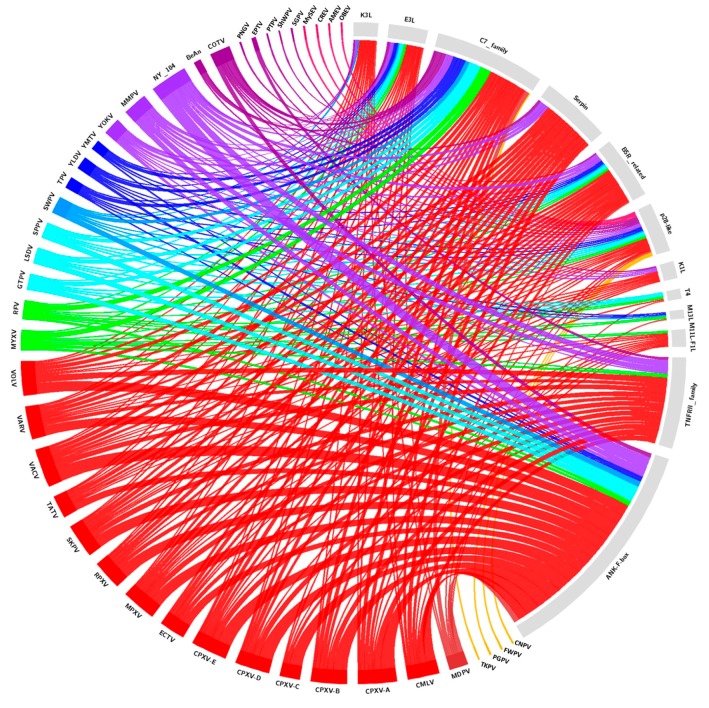
Circos plot representing the amount and diversity of host range genes among the poxviruses. Genomic information was available at the NCBI database, following the established criteria (E-value ≤ 0.0001; Ident ≥ 30%; Coverage ≥ 40%). The different colors stand for the genus of poxviruses and the family of host range genes. The full names of the viruses are listed in Supplementary Table S1.

We reviewed the complete genomic sequences available at the National Center for Biotechnology Information database (https://www.ncbi.nlm.nih.gov) for 38 identified and putative homologs of poxvirus host range genes, and updated this gene data with new deposited poxvirus sequences. The searches for poxvirus host range gene homologs were performed using Basic Local Alignments Search Tools—Protein (BLASTP, https://blast.ncbi.nlm.nih.gov/Blast.cgi). Protein sequences of previously identified host range genes were used as reference sequences for BLASTP analyses (Supplementary Table S3). For the analysis, putative homologs were considered inside of a fixed cutoff threshold for all genes (E-value ≤ 0.0001; Ident ≥ 30%; Coverage ≥ 40%). Using the obtained data, a circle plot was created (Figure 2), indicating the presence of homologous genes in different species and samples of poxviruses.

**Figure 3:**
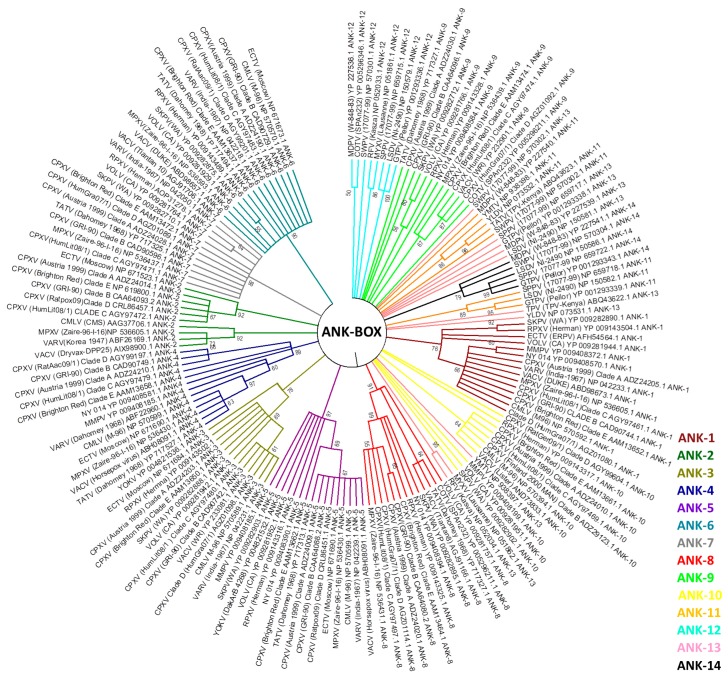
Presence of Ank-box host range gene projected onto a tree showing the phylogenetic relationships of representative poxvirus strains. The tree was generated from a multiple protein sequence alignment of the indicated sequences using the maximum likelihood method. A bootstrap of 1000 was used, and support >50 is indicated above the branches.

**Figure 4:**
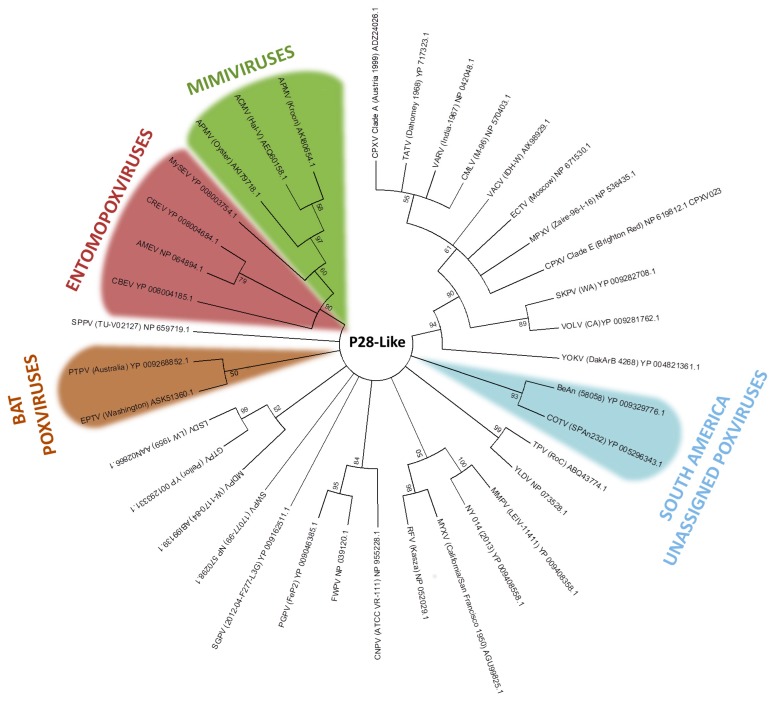
Phylogenetic relationship of p28-like genes. The tree was generated from a multiple protein sequence alignment of the indicated sequences using the maximum likelihood method. A bootstrap of 1000 was used, and support >50 is indicated above the branches.

**Figure 5:**
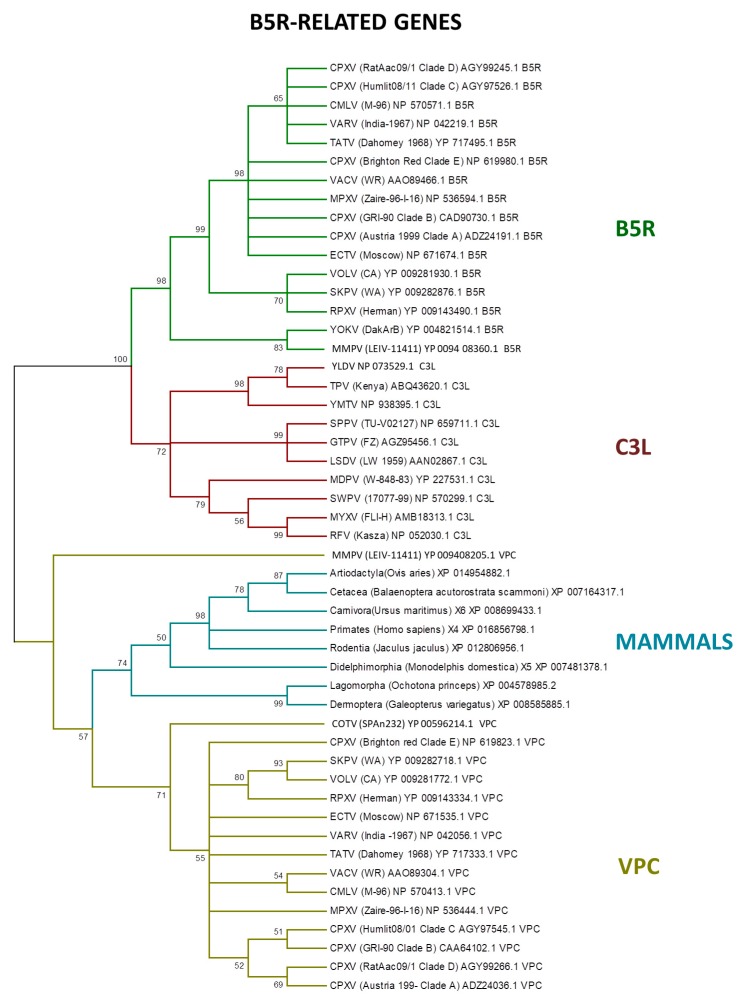
Phylogenetic relationship of poxvirus B5R-related proteins. The tree was generated from a multiple protein sequence alignment of the indicated sequences using the maximum likelihood method. A bootstrap of 1000 was used, and support > 50 is indicated above the branches.

Analyzed homologs are found in the genera *Orthopoxvirus* (28), *Leporipoxvirus* (14), *Cervidpoxvirus* (13), *Suipoxvirus* (12), *Capripoxvirus* (11), *Centapoxvirus* (11), *Yatapoxvirus* (9), *Avipoxvirus* (3)—including Shearwater poxvirus (ShWPV) and Penguin poxvirus (PNGV)—in the unassigned species *Pteropox virus* (1), and in the unassigned samples NY_014 poxvirus (NY_014) (26), Murmansk poxvirus (MMPV) (17), Cotia virus (COTV) (13), Yaba-like disease virus (YLDV) (8), BeAn58058 (BeAn) (5) and Eptesipox virus (ETPV) (3). Within the estimated cut-offs, homologs were not found in the *Crocodylidpoxvirus*, *Molluscipoxvirus*, or *Parapoxvirus,* nor all of the entomopoxviruses genera. It is noteworthy that the CPXV species has up to 27 host range gene homologs; however, when we analyze the different isolates as separate clades, groups A (27), B (26), D (25), and E (25) have very close numbers of homologs, while group C (14) presented considerably fewer homologs. On the other hand, phylogenetic analyses of various host range genes show that different clades of CPXV are sometimes grouped with another OPV species rather than each other. These data are in accordance with the current hypotheses about diversity within CPXV species (Figure 3, Figure 4 and Figure 5, Supplementary Figures S1 and S2) [44]. The genes that have homologs with the highest range of species are E3L, C7L, and m62r (C7L Family) with 25, 24, and 19 viral species, respectively (Figure 2). One Ank-box homolog and the F1L are unique to the genus *Orthopoxvirus* (Figure 2). M11L and m63r (C7L Family) were found only in *Leporipoxvirus*. M11L and F1L-encoded proteins were related to the cellular, prosurvival Bcl-2 family, but there was not any primary sequence similarity in our searches and previous works [20]. Our data corroborates with the previously described literature concerning the distribution of host range families in poxvirus genera, since orthopoxviruses and clade II poxviruses contain the majority of homologs (Figure 3; Supplementary Figures S1 and S2) [17,20,21]. One factor that could influence this is the concentration of studies in the OPV and clade II viruses. Thus, the distantly related poxviruses likely contain different genes that are important for their host range, but have not yet been identified.

Another important point to be addressed is that no clear correlation was observed in the host range size versus the host range gene homologs’ diversity and quantity. For instance, *Parapoxvirus* (ORFV, PCPV, BPSV, *Parapoxvirus of red deer in New Zealand*—PRDV) is associated with a host range that is equal to or larger than other poxvirus groups, which have a wide range of host gene homologs (Figure 1 and Figure 2). However, parapoxviruses have a strict range of permissive cells, including primary ovine and bovine fibroblasts [18]. Two other viruses that clearly show the absence of direct correlation between the host range and host range genes are MOCV and VARV. Both viruses have the narrowest tropism of any poxvirus, and use humans as exclusive natural hosts [18]. The MOCV genome lacks more than 80 genes common to most orthopoxviruses, including VARV [87]. These genes have a functional role in the suppression of the host response to viral infection, and their absence may be related to MOCV’s restricted replication in basal keratinocytes of the human epidermis [21,87]. In contrast, VARV’s replication range includes most mammalian cells [18]. Although the VARV host range is remarkably different from other OPVs—such as CPXV, VACV, and MPXV—in terms of their reservoir or zoonotic hosts, in tissue culture cells, these differences disappear, and the host range genes influence this homogeneity within the genus. Although MOCV and VARV only infect humans, the presence or absence of different host range genes may be related to different means of evolution, and culminate in different pathogenesis, tropism, and immune regulation between both viruses. Ecological aspects of virus–host relationships such as host abundance, environmental influences on the transmission cycle, geographical distribution, and overlapping areas of species may be essential for the dynamics of the host spectrum of a virus. Take for example the globalized geographical aspect of myxomatosis in Australian rabbits, whereby a South American virus infects a European host in a new continent for both species, i.e., virus and host. Taking these aspects into account, the current view of the poxviruses’ host range can be highly complex and could contain some biased points. There are plenty of unanswered questions relative to the host range genes and the host spectrum of poxviruses, and ecological and evolutionary perspectives may provide important insights about this.

## 3. Host Range Genes: An Evolutionary Perspective

The evolution of virulence genes for a certain host is probably influenced by the effectiveness and route of virus transmission, the immune status of the host species, the availability of additional reservoir hosts, intra- and inter-species competition with other viruses, and positive selection for hosts that are more resistant to virus infection [19,88]. The origin of viruses, or at least certain viral groups, is still a mystery. It is possible that some viral groups have a degree of monophyleticism. The NCLDV group, which includes the family *Poxviridae* as well as other large dsDNA viruses that infect eukaryotes, is a candidate for monophyly [89,90]. In phylogenetic analyses, there is evidence of an ancient origin provided by a topology in which all of the poxvirus homologs cluster together, apart from those of cellular organisms [16,91]. Certain viral-specific genes may be involved in poxvirus evolutionary processes, including some host range genes.

In this study, we review this evidence in three aspects of host range genes in poxvirus evolutionary biology: (1) the phylogeny of previously identified poxvirus host range genes; (2) possible lateral transfer of host range genes; and (3) evolutionary correlations with other viral groups. We used sequence alignments containing the homologous protein sequences of 38 identified host range genes. A total of 396 completely sequenced poxvirus genomes available in public databases were used to perform phylogenetic analyses. These sequences belong to genera *Avipoxvirus* (8), *Capripoxvirus* (15), *Centapoxvirus* (3), *Cervidpoxvirus* (2), *Crocodylidpoxvirus* (1), *Leporipoxvirus* (64), *Molluscipoxvirus* (5), *Orthopoxvirus* (262), *Parapoxvirus* (17), *Suipoxvirus* (1), *Yatapoxvirus* (4), entomopoxviruses (7), and also unassigned species and isolates (7). Most of the chordopoxviruses genomes are concentrated in the genera *Orthopoxvirus* and *Leporipoxvirus,* although at least one genome is available by genus. The sequence alignment was performed using ClustalW [92] to infer a maximum likelihood phylogenetic tree. In our phylogenetic analyses, the E3L, C7L, and Ank-Box host range genes supported the evolutionary correlation between OPV and clade II poxviruses (Figure 3; Supplementary Figures S1 and S2). Murmansk poxvirus and NY 014 poxvirus samples were closely related to YOKV, which are likely unclassified centapoxviruses [93]. The Cotia virus is grouped within poxvirus Clade II, as described above, and the unknown samples BeAn58058 follow this trend. In addition, ANK repeat proteins were highly abundant in various poxvirus genera. The OPV ANK-box homologs have similarities with those found in YOKV, NY 014 poxvirus, Murmansk poxvirus, and Cotia virus. Clade II Ank-box homologs are present in *Leporipoxvirus* (2 homologs), *Cervidpoxvirus* (4 homologs), *Suipoxvirus* (4 homologs), *Capripoxvirus* (4 homologs), *Yatapoxvirus* (2 homolog), Yaba-like disease virus (2) and Cotia virus (1) (Figure 3). 

Between the 14 ANK/F-box homolog genes analyzed, none were similar to ANK, which was described in the literature for avipoxvirus and parapoxvirus. This indicated a distant correlation between these homologs and those of other poxviruses [20]. ANK/F-Box genes probably originated from multiple duplications in all poxviruses groups, and our data suggested that these duplications occurred after the radiation of orthopoxviruses and clade II poxviruses of another family of viruses [20].

The p28/N1R RING zinc finger protein is the most abundant gene family in poxviruses, and has also been identified in other NCLDVs, such as iridoviruses and mimiviruses [94,95]. In our analysis, p28-like protein homologs are found in most members of the *Chordopoxvirinae* subfamily and in acanthamoeba polyphaga mimivirus (*Mimiviridae*), as previously described [96]. This fact may reinforce the common ancestry hypothesis between both families. The recently discovered chordopoxvirus Salmon gill poxvirus (SGPV), appears to be a distant chordopoxvirus [97]. The p28-like protein homologs of Cotia virus and BeAn58058 form a high bootstrap clade (Figure 4). Despite Eptesipox virus forming a well-supported clade with Pteropox virus, both viruses were isolated from bats, which could suggest an adaptation of p28 to the host [98,99]. However, p28-like protein homologs of entomopoxvirus and mimivirus formed an external clade (Figure 4). Even considering the ancestry of these groups, the sharing of such genes across distinct viral groups might in turn be explained by LGT events [97].

LGT is an important process in viral evolution, and in pathogenic viruses, this process can often increase their virulence and fitness [16,17,96,100,101,102,103]. Poxviruses also express proteins homologous to the vertebrate immune system signaling molecules or receptors, which are encoded by genes that were probably incorporated from the host [91]. Among the host range genes, the serpin family members are potential candidates to LGT, as identified by PSI–BLAST search [15,104]. In addition, the B5R-related genes express CD46-like proteins (30–40% similar with mammal hosts) to control the host complement system [105,106]. Our analyses showed that the B5R-related genes are closely related with the homologs of several mammalian orders (Figure 5).

Previous studies indicated that the ancestral B5R/C3L genes faced a duplication event early in orthopoxvirus evolution [20]. Indeed, the B5R gene retained the transmembrane domain, keeping the protein trapped in the external enveloped virion membrane, while the other homologs—VCP and C3L—are secreted, which is an example of neofunctionalization as a different virulence factor by inhibiting the host complement factors [78,107,108].

## 4. Future Directions

The poxviruses are one of the most intensively studied groups among the virosphere, especially because of their medicinal importance for humans and a large range of domesticated animals. These viruses have been isolated and identified in different groups of animals, both vertebrates and invertebrates, and it has been tempting to correlate this host spectrum to the amount and diversity of the so-called host range genes [18,19,20]. Although some host range genes were described in viruses with numerous hosts, such as CPXV, no apparent correlations between the host organisms and the number and diversity of host range genes have been found. Furthermore, the high number of these genes described in VACV, CPXV, and MPXV may be directly related to the large number of studies about these viruses. In addition, viruses with the same host range, such as VARV and MOCV, do not share the same host range genes. On the other hand, the presence of host range genes is better viewed and characterized when referring to the range of cells permissive to viral replication, thus having little effect (if any) on the capacity of a virus to infect an animal host.

The evolutionary origins of poxviruses’ host range gene families are likely diverse. Some genes are ancient, and remain conserved in several groups of NCLDVs, while others probably evolved due to lineage-specific duplications. LGT events are also possible between different viruses and between viruses and their hosts. The description of new poxvirus samples, especially in previously unrelated hosts, such as those recently discovered in bats and fish, can bring new aspects of poxvirus biology and new host range genes. Other genes in these families are likely to possess host range functions that might impact the host spectrum of viruses at the organism level. These analyses will also provide valuable insights about the risk assessment for poxvirus emergence. In light of the data presented here, great effort must be taken to better elucidate the poxvirus host network, especially concerning the entomopoxviruses. Furthermore, it seems that the term ‘host range genes’ is not the most appropriate for these virulence genes, since there is no direct correlation between their presence and the host range of poxviruses, at least when considering animals as hosts, rather than cells. More in-depth investigation regarding the diversity of the poxvirus and their hosts, associated with in silico, in vitro, and in vivo assays about these virulence genes, will certainly bring more insights into this intriguing field of virus–host coevolution.

## Supplementary Materials

The following are available online at www.mdpi.com/1999-4915/9/11/331/s1, Figure S1: Phylogenetic relationship of C7L gene. The tree was generated from a multiple protein sequence alignment of the indicated sequences using the maximum likelihood method. A bootstrap of 1000 was used, and support >50 is indicated above the branches. Branches in red represent the OPV clade, and branches in green represent clade II poxviruses; Figure S2: Phylogenetic relationship of E3L gene. The tree was generated from a multiple protein sequence alignment of the indicated sequences using the maximum likelihood method. A bootstrap of 1000 was used, and support >50 is indicated above the branches. Branches in red represent the OPV clade, and branches in green represent clade II poxviruses; Table S1: Names and abbreviations of the International Committee on Taxonomy of Viruses (ICTV) poxviruses species; Table S2: Poxvirus species and the associated hosts; Table S3: Protein sequences used as references for the BLASTP analyses.
