# Supplementary material for: Poxvirus Host Range Genes and Virus–Host Spectrum: A Critical Review
Source: Viruses. 2017 Nov 7;9(11):331. doi: 10.3390/v9110331 (PMC5707538; doi:10.3390/v9110331)
Supplement: Supplementary file 1 [file viruses-09-00331-s001.zip › viruses-231207-supplementary/Sup table 1.pdf]

**Chordopoxvirinae**		
**Name**	**Abbreviation**	**Genus**
Fowlpox virus	FWPV	*Avipoxvirus*
Psittacinepox virus	PTCV	*Avipoxvirus*
Mynahpox virus	MNPV	*Avipoxvirus*
Sparrowpox virus	SPWV	*Avipoxvirus*
Starlingpox virus	SLPV	*Avipoxvirus*
Canarypox virus	CNPV	*Avipoxvirus*
Juncopox virus	JCPV	*Avipoxvirus*
Quailpox virus	QLPV	*Avipoxvirus*
Pigeonpox virus	PGPV	*Avipoxvirus*
Turkeypox virus	TKPV	*Avipoxvirus*
Shearwater poxvirus	ShWPV	Avipoxvirus (putative)
Penguin poxvirus	PNGV	Avipoxvirus (putative)
Goatpox virus	GTPV	*Capripoxvirus*
Lumpy skin disease virus	LSDV	*Capripoxvirus*
Sheeppox virus	SPPV	*Capripoxvirus*
Yokapox virus	YOKV	*Centapoxvirus*
NY_014 poxvirus	NY_014	Centapoxvirus (putative)
Murmansk poxvirus	MMPV	Centapoxvirus (putative)
Mule deerpox virus	MDPV	*Cervidpoxvirus*
Nile crocodilepox virus	NCPV	*Crocodylidpoxvirus*
Myxoma virus	MYXV	*Leporipoxvirus*
Rabbit fibroma virus	RFV	*Leporipoxvirus*
Squirrel fibroma virus	SFV	*Leporipoxvirus*
Hare fibroma virus	HFV	*Leporipoxvirus*
Molluscum contagiosum virus	MOCV	*Molluscipoxvirus*
Ectromelia virus	ECTV	*Orthopoxvirus*
Skunkpox virus	SKPV	*Orthopoxvirus*
Camelpox virus	CMLV	*Orthopoxvirus*
Cowpox virus	CPXV	*Orthopoxvirus*
Raccoonpox virus	RPXV	*Orthopoxvirus*
Taterapox virus	TATV	*Orthopoxvirus*
Vaccinia virus	VACV	*Orthopoxvirus*
Variola virus	VARV	*Orthopoxvirus*
Volepox virus	VOLV	*Orthopoxvirus*
Orf virus	ORFV	*Parapoxvirus*
Parapoxvirus of red deer in New Zealand	PRDV	*Parapoxvirus*
Pseudocowpox virus	PCPV	*Parapoxvirus*
Bovine papular stomatitis virus	BPSV	*Parapoxvirus*
Swinepox virus	SWPV	*Suipoxvirus*
Squirrelpox virus	SQPV	Unassigned
Pteropox virus	PTPV	Unassigned
Cotia virus	COTV	Unassigned
BeAn58058	BeAn	Unassigned
Eptesipox virus	ETPV	Unassigned
Salmon Gill Poxvirus	SGPV	Unassigned
Tanapox virus	TPV	*Yatapoxvirus*
Yaba monkey tumor virus	YMTV	*Yatapoxvirus*
Yaba-like disease virus	YLDV	Yatapoxvirus (putative)

**Entomopoxvirinae**		
**Name**	**Abbreviation**	**Genus**
Aphodius tasmaniae entomopoxvirus	ATEV	*Alphaentomopoxvirus*
Geotrupes sylvaticus entomopoxvirus	GSEV	*Alphaentomopoxvirus*
Anomala cuprea entomopoxvirus	ACuEV	*Alphaentomopoxvirus*
Melolontha melolontha entomopoxvirus	MMEV	*Alphaentomopoxvirus*
Demodema bonariensis entomopoxvirus	DBEV	*Alphaentomopoxvirus*
Dermolepida albohirtum entomopoxvirus	DAEV	*Alphaentomopoxvirus*
Figulus sublaevis entomopoxvirus	FSEV	*Alphaentomopoxvirus*
Choristoneura fumiferana entomopoxvirus	CFEV	*Betaentomopoxvirus*
Mythimna separata entomopoxvirus	MySEV	*Betaentomopoxvirus*
Locusta migratoria entomopoxvirus	LMEV	*Betaentomopoxvirus*
Arphia conspersa entomopoxvirus	ACEV	*Betaentomopoxvirus*
Choristoneura diversuma entomopoxvirus	CDEV	*Betaentomopoxvirus*
Chorizagrotis auxiliaris entomopoxvirus	CAuEV	*Betaentomopoxvirus*
Choristoneura rosaceana entomopoxvirus	CREV	*Betaentomopoxvirus*
Heliothis armigera entomopoxvirus	HAEV	*Betaentomopoxvirus*
Oedaleus senegalensis entomopoxvirus	OSEV	*Betaentomopoxvirus*
Adoxophyes honmai entomopoxvirus	AHEV	*Betaentomopoxvirus*
Amsacta moorei entomopoxvirus	AMEV	*Betaentomopoxvirus*
Choristoneura biennis entomopoxvirus	CBEV	*Betaentomopoxvirus*
Choristoneura conflicta entomopoxvirus	CCEV	*Betaentomopoxvirus*
Acrobasis zelleri entomopoxvirus	AZEV	*Betaentomopoxvirus*
Operophtera brumata entomopoxvirus	OBEV	*Betaentomopoxvirus*
Schistocerca gregaria entomopoxvirus	SGEV	*Betaentomopoxvirus*
Aedes aegypti entomopoxvirus	AEEV	*Gammaentomopoxvirus*
Chironomus attenuatus entomopoxvirus	CAtEV	*Gammaentomopoxvirus*
Goeldichironomus holoprasinus entomopoxvirus	GHEV	*Gammaentomopoxvirus*
Chironomus luridus entomopoxvirus	CLEV	*Gammaentomopoxvirus*
Camptochironomus tentans entomopoxvirus	CTEV	*Gammaentomopoxvirus*
Chironomus plumosus entomopoxvirus	CPEV	*Gammaentomopoxvirus*
Diachasmimorpha entomopoxvirus	DMEV	Unassigned
Melanoplus sanguinipes entomopoxvirus 'O'	MSEV	Unassigned
