# Supplementary material for: Poxvirus Host Range Genes and Virus–Host Spectrum: A Critical Review
Source: Viruses. 2017 Nov 7;9(11):331. doi: 10.3390/v9110331 (PMC5707538; doi:10.3390/v9110331)
Supplement: Supplementary file 1 [file viruses-09-00331-s001.zip › viruses-231207-supplementary/Sup table 3.pdf]

Gene Group	Host Range Gene	Protein Reference Sequence
K3L	K3L	VACV-WR [034] (AAO89313.1)
E3L	E3L	VACV-WR [059] (AAO89338.1)
Serpins	SPI-1	VACV-WR [205] (AAO89484.1)
	SPI-2	VACV-WR [195] (AAO89474.1)
	SPI-3	VACV-WR [033] (AAO89312.1)
p28-like	p28-like	RFV Rabbit fibroma virus[s143R] (NP_052029.1)
	p28-like	CPXV [023] (NP_619812.1)
	p28-like	CNPV [103] (NP_955126.1)
	p28-like	SGPV (YP_009162511.1)
	p28-like	APMV (AKI80654.1)
C7 family	C7L	VACV-WR [021] (AAO89300.1)
	C4L	CPXV-GRI90 [C4L] (CAD90593.1)
	M062R	MYXV-FLIH [m062R] (AMB18231.1)
	M063R	MYXV-FLIH [m062R] (AMB18232.1)
	M064R	MYXV-FLIH [m062R] (AMB18233.1)
T4	T4	MYXV-FLIH [m004R] (AMB18330.1)
B5R-related	B5R	VACV-WR [187] (AAO89466.1)
	VCP	VACV-WR [025] (AAO89304.1)
	C3L	MYXV-FLIH [m144R] (AMB18313.1)
M13L	M13L	MYXV-FLIH [m013L] (AMB18183.1)
M11L/F1L	M11L	VACV-WR [040] (AAO89319.1)
	F1L	MYXV-FLIH [m011L] (AMB18181.1)
K1L	K1L	VACV-WR [032] (AAO89311.1)
TNFR II family	T2	MYXV-MAV [m002R] (AMB18488.1)
	CrmB	CPXV-Brighton Red [005] (AAA60952.1)
	CrmC	CPXV-Brighton Red [191] (AAM13631.1)
	CrmD	CPXV-Brighton Red [221] (AAM13659.1)
	CrmE	CPXV-EV(CAC15562.1)
ANK/F-box	Ank 1	CPXV-Brighton Red [213] (AAM13652.1)
	Ank 2	CPXV-Brighton Red [011] (AAM13458.1)
	Ank 3	CPXV-Brighton Red [211] (AAM13650.1)
	Ank 4	CPXV-Brighton Red [220] (AAM13658.1)
	Ank 5	CPXV-Brighton Red [225] (AAM13662.1)
	Ank 6	CPXV-Brighton Red [198] (AAM13637.1)
	Ank 7	CPXV-Brighton Red [025] (AAM13472.1)
	Ank 8	CPXV-Brighton Red [017] (AAM13464.1)
	Ank 9	CPXV-Brighton Red [027] (AAM13474.1)
	Ank 10	CPXV-Brighton Red [223] (AAM13661.1)
	Ank 11	DPV-W-848-83 [165] (YP_227540.1)

Ank 12	DPV-W-848-83 [161] (YP_227536.1)
Ank 13	DPV-W-848-83 [164] (YP_227539.1)
Ank 14	DPV-W-848-83 [166] (YP_227541.1)
