# Supplementary figures and images for: Poxvirus Host Range Genes and Virus–Host Spectrum: A Critical Review
Source: Viruses. 2017 Nov 7;9(11):331. doi: 10.3390/v9110331 (PMC5707538; doi:10.3390/v9110331)

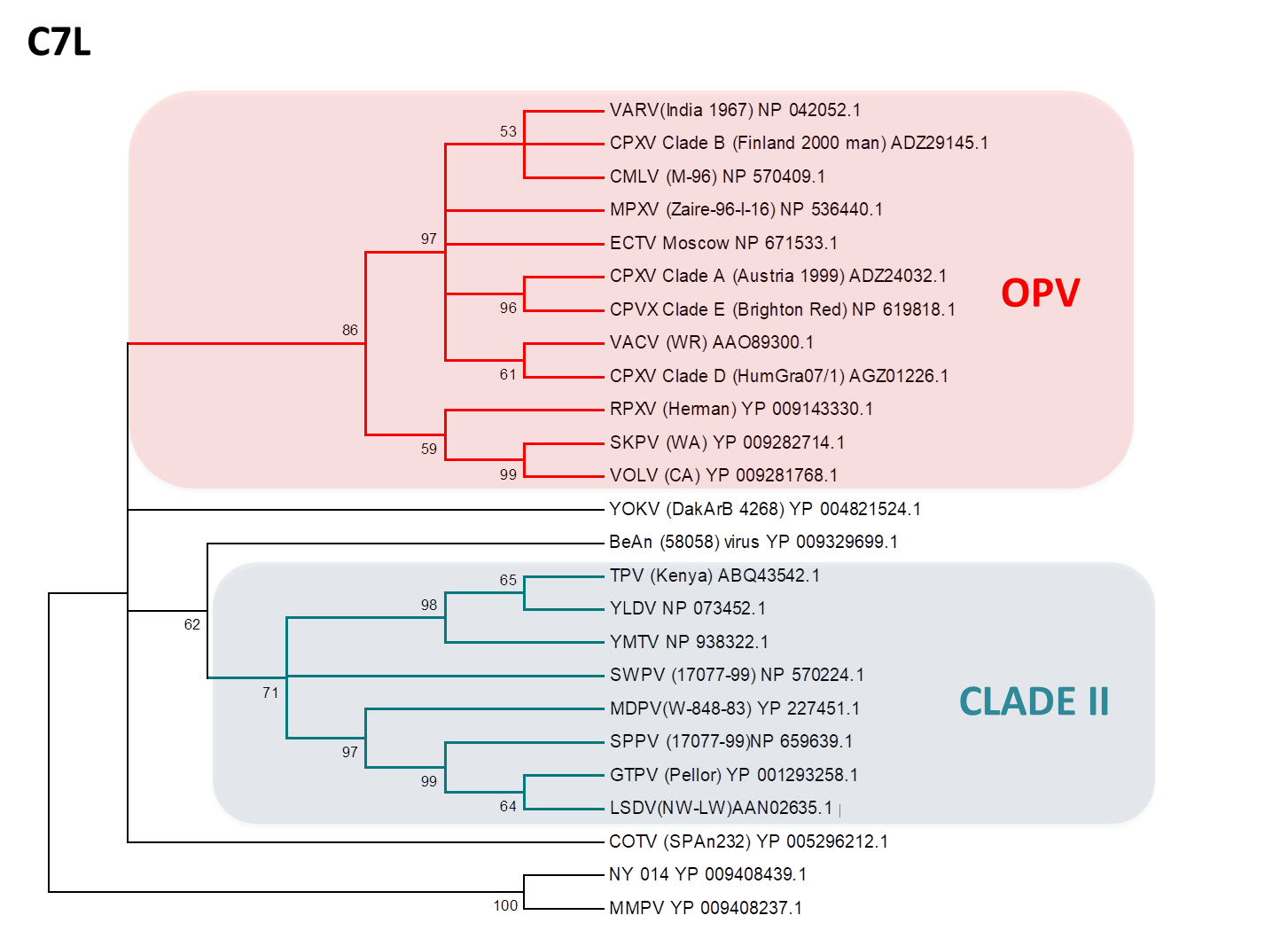

Supplement: Supplementary file 1 [file viruses-09-00331-s001.zip › viruses-231207-supplementary/Sup Figure 1..jpg]

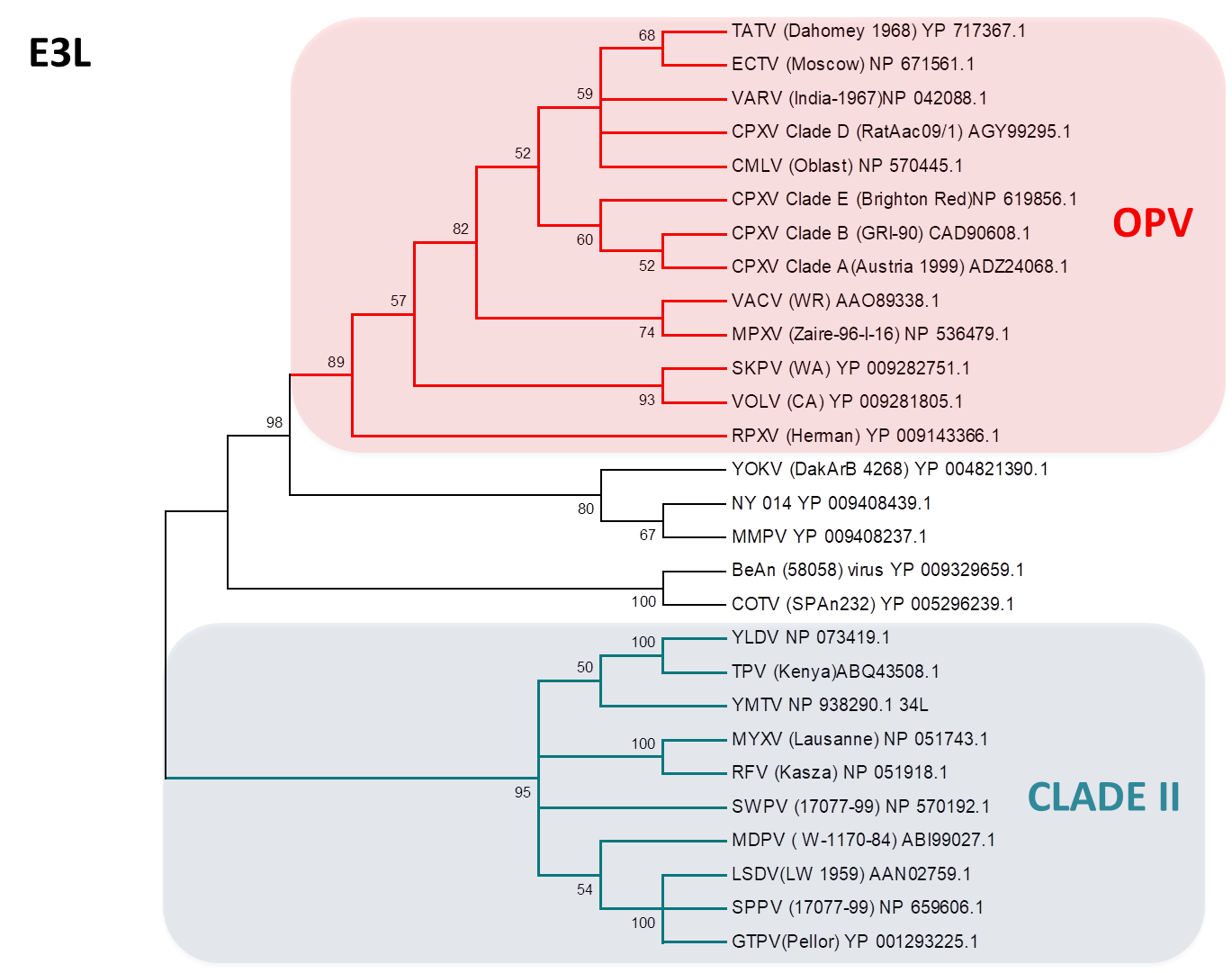

Supplement: Supplementary file 1 [file viruses-09-00331-s001.zip › viruses-231207-supplementary/Sup Figure 2.jpg]
