# Supplementary material for: Poxvirus Host Range Genes and Virus–Host Spectrum: A Critical Review
Source: Viruses. 2017 Nov 7;9(11):331. doi: 10.3390/v9110331 (PMC5707538; doi:10.3390/v9110331)
Supplement: Supplementary file 1 [file viruses-09-00331-s001.zip › viruses-231207-supplementary/Sup. Figures Captions.doc]

**Supplementary Figure 1: Phylogenetic relationship of C7L gene.** The tree was generated from a multiple protein sequence alignment of the indicated sequences using the maximum likelihood method. A bootstrap of 1,000 was used, and support >50 is indicated above the branches. Branches in red represent the OPV clade, and branches in green represent clade II poxviruses.

**Supplementary Figure 2: Phylogenetic relationship of E3L gene.** The tree was generated from a multiple protein sequence alignment of the indicated sequences using the maximum likelihood method. A bootstrap of 1,000 was used, and support >50 is indicated above the branches. Branches in red represent the OPV clade, and branches in green represent clade II poxviruses.

**Supplementary Table 1:** Names and abbreviations of the ICTV poxviruses species.

**Supplementary Table 2:** Poxvirus species and the associated hosts.

**Supplementary Table 3:** Protein sequences used as references for the BLASTP analyses.
